# 866. Adherence to F/TDF for PrEP in Dried Blood Spots and HIV Infection Rates: A Pooled Analysis of Global PrEP Studies

**DOI:** 10.1093/ofid/ofab466.1061

**Published:** 2021-12-04

**Authors:** Albert Liu, Albert Liu, Robert Grant, Raphael J Landovitz, Raphael J Landovitz, Beatriz Grinsztejn, Connie Celum, Jared Baeten, David Magnuson, Moupali Das, Christoph C Carter, Dawn Smith, Li Tao

**Affiliations:** 1 Bridge HIV, San Francisco Department of Public Health, CA; 2 University of California, San Francisco, San Francisco, CA; 3 UCLA Center for Clinical AIDS Research & Education, Los Angeles, CA; 4 15. Instituto Nacional de Infectologia Evandro Chagas, Fundação Oswaldo Cruz, Rio de Janiero, Rio de Janeiro, Brazil; 5 University of Washington, Seattle, Washington; 6 Gilead Sciences Inc., Foster City, CA; 7 Division of HIV/AIDS Prevention (DHAP), Atlanta, Georgia; 8 Gilead Science, Inc, Foster City, CA

## Abstract

**Background:**

The use of daily F/TDF for HIV pre-exposure prophylaxis (PrEP) substantially reduces HIV acquisition. Dried blood spot (DBS) tenofovir-diphosphate (TFV-DP) levels reflect TDF use over the past 6-8 weeks, providing an objective measure of adherence in people taking PrEP.

**Methods:**

In a pooled analysis of 19 PrEP demonstration projects and clinical studies, 6,613 participants had at least one TFV-DP measurement in DBS and followed for at least 48 weeks and up to 96 weeks. We used a piecewise linear mixed-effects model to plot the least-square means with corresponding 95% confidence intervals (CI) of TFV-DP for adherence over time, and Poisson regressions to calculate HIV incidence rates (IR) by level of weighted average of TFV-DP.

**Results:**

Of 6,613 participants, median age was 30 years (interquartile range 24−38), 5,449 (82%) were cisgender men, 806 (12%) were cisgender women, and 349 (5%) were transgender (316 transgender women, 2 transgender men, 31 unspecified). Adherence based on TFV-DP in DBS was consistently higher among participants who did not acquire HIV compared to those who did (Figure). Among all participants, 21%, 14%, 36%, and 29% has DBS consistent with taking < 2, 2−3, 4−6, and ≥7 tablets of F/TDF PrEP per week (Table). Sixty-nine participants acquired HIV, with a median PrEP exposure of 0.82 years and an overall HIV IR (95% CI) of 1.16 (0.92, 1.47) per 100 person years. There was a strong association between adherence and HIV incidence [among individuals who took < 2, 2−3, 4−6, and ≥7 tablets/week, the HIV IRs (95% CI) were 5.20 (4.03, 6.71), 0.38 (0.12, 1.18), 0.28 (0.12, 0.61), and 0.06 (0.01, 0.39), respectively. Overall IR (95% CI) of HIV infection among cisgender men was 1.25 (0.98, 1.60) per 100 patient-years. Four cisgender women and 2 transgender participants acquired HIV, corresponding to IRs (95% CI) of 0.71 (0.27, 1.90) and 0.63 (0.16, 2.53).

Adherence by TFV-DP in DBS for F/TDF users who acquired HIV compared to those who did not.

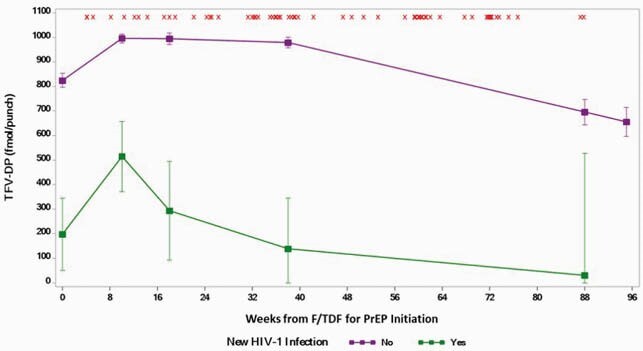

Note: ‘x’ on the Figure represents visit week when a new HIV infection was detected.

HIV incidence rates (95% confidence intervals) by adherence to PrEP measured by level of TFV-DP in DBS up to 96 weeks after PrEP Initiation

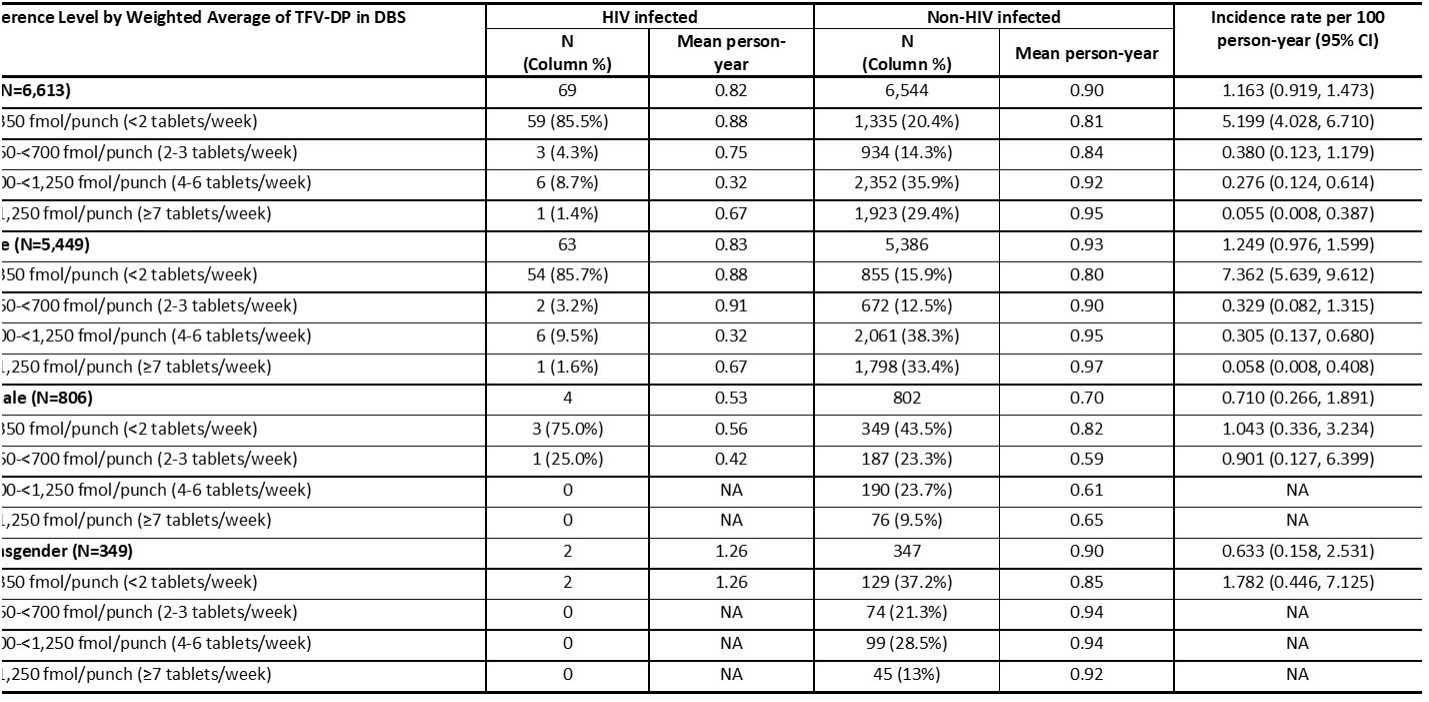

**Conclusion:**

This diverse, multi-national pooled analysis of F/TDF PrEP use provides the largest assessment to date of the adherence-HIV incidence relationship in people taking F/TDF for PrEP. The results suggest a high background HIV incidence in the pooled cohort and high efficacy in those adherent to PrEP. These findings support ongoing efforts to increase PrEP use among people who would benefit.

**Disclosures:**

**Albert Liu, MD, MPH**, Gilead Sciences (Individual(s) Involved: Self): Gilead has donated study drug for studies I have led., Grant/Research Support, Other Financial or Material Support, Research Grant or Support; IAS-USA (Individual(s) Involved: Self): Honorarium for manuscript writing, Other Financial or Material Support; Viiv Healthcare (Individual(s) Involved: Self): Grant/Research Support, Research Grant or Support **Raphael J. Landovitz, MD, MSc**, Gilead Sciences (Individual(s) Involved: Self): Consultant; Janssen (Individual(s) Involved: Self): Consultant; Merck Inc (Individual(s) Involved: Self): Consultant; Roche (Individual(s) Involved: Self): Consultant **Jared Baeten, MD, PHD**, **Gilead Sciences Inc.** (Employee, Shareholder) **David Magnuson, PharmD**, **Gilead Sciences Inc** (Employee, Shareholder) **Moupali Das, MD**, **Gilead Sciences Inc.** (Employee, Shareholder) **Christoph C. Carter, MD**, **Gilead Sciences Inc.** (Employee, Shareholder) **Li Tao, MD, PhD**, **Gilead Sciences Inc** (Employee, Shareholder)

